# Multiple stakeholders' perspectives on patient and public involvement in community mental health services research: A qualitative analysis

**DOI:** 10.1111/hex.13529

**Published:** 2022-06-03

**Authors:** Sosei Yamaguchi, Makiko Abe, Takayuki Kawaguchi, Momoka Igarashi, Takuma Shiozawa, Makoto Ogawa, Naonori Yasuma, Sayaka Sato, Yuki Miyamoto, Chiyo Fujii

**Affiliations:** ^1^ Department of Community Mental Health and Law, National Institute of Mental Health National Center of Neurology and Psychiatry Kodaira Tokyo Japan; ^2^ Department of Psychiatric Nursing, Graduate School of Medicine The University of Tokyo Bunkyo Tokyo Japan

**Keywords:** community mental health, focus group interview, mental health services research, patient and public involvement, qualitative analysis

## Abstract

**Background:**

Patient and public involvement (PPI) has become essential in health research. However, little is known about multiple stakeholders' perspectives on the implementation of PPI in community mental health research settings. The present study aimed to qualitatively analyse multiple stakeholders' views on PPI, including potential concerns, barriers and approaches.

**Methods:**

This study involved conducting focus group interviews and collecting qualitative data from 37 participants in multiple stakeholder groups (patients = 6, caregivers = 5, service providers = 7, government staff = 5 and researchers = 14) in the community mental health field. The data were qualitatively analysed using a data‐driven approach that derived domains, themes and subthemes related to perspectives on PPI and to specific challenges and approaches for implementing PPI.

**Results:**

The qualitative analysis identified four domains. The ‘Positive views and expectations regarding PPI’ domain consisted of themes related to supportive views of PPI in a mental health service research setting and improvements in the quality of research and service. The ‘General concerns about PPI’ domain included themes concerning the need for non‐PPI research and tokenism, excessive expectations concerning social changes and use of evidence from PPI research, and heavy burdens resulting from PPI. The ‘Specific issues regarding the implementation of PPI’ domain consisted of four themes, including academic systems, selection methods (e.g., representativeness and conflict of interest issues), relationship building, and ambiguous PPI criteria. In particular, all stakeholder groups expressed concerns about relational equality during PPI implementation in Japan. The ‘Approaches to PPI implementation’ domain included themes such as facilitating mutual understanding, creating a tolerant atmosphere, establishing PPI support systems (e.g., training, ethics and human resource matching) and empowering patient organizations.

**Conclusion:**

The study replicated most of the barriers and approaches to PPI reported by qualitative research in Western counties. However, utilization of evidence produced by PPI research and partnership in the PPI process may be particularly serious issues in Japan. Future PPI studies should carefully address solutions that fit each culture.

**Patient or Public Contribution:**

A patient‐researcher was involved in all stages of this project, from development of the research topic and the protocol to manuscript preparation.

## 1 INTRODUCTION

Patient and public involvement (PPI) has become essential in health service research. PPI refers to the involvement of laypersons in research, including patients, caregivers and other community members. A definition of PPI is ‘research being carried out “with” or “by” members of the public rather than “to”, “about” or “for” them’.^1^ The value of research collaboration with patients has been gradually recognized and disseminated in research on mental health services and community services.^2^, ^3^ However, PPI in research on community mental health services is not widespread in non‐Western countries. In this paper, based on recent discussions, we refer to those who have been diagnosed with a mental illness or who use mental health services as patients rather than service users.^4^, ^5^

There are several benefits and barriers to implementing PPI in research. For example, several systematic reviews reported that PPI potentially contributed towards identifying high‐priority research topics, selecting appropriate outcomes and ethical data collection methods, creating patient‐friendly study materials, increasing participant enrolment rates and facilitating interpretation of results from a patient perspective.^6^, ^7^, ^8^, ^9^, ^10^, ^11^, ^12^, ^13^, ^14^ In addition, PPI may empower patients and provide researchers with opportunities to increase patient insights into individual research fields.^15^ Conversely, reviews have identified barriers to PPI implementation, including increased research cost and duration, tokenistic adoption, burdens on both patients and researchers, inequality between patients and researchers and a lacks of researchers' communication skills, the need to employ a researcher defined as an expert by experiencing living with mental illness (patient‐researcher or user‐researcher) and inadequate relationship building.^10^, ^16^, ^17^, ^18^, ^19^ In summary, previous studies have identified the advantages and drawbacks of PPI in research.

Despite a large body of literature on PPI, additional research is needed in the community mental health service field. Strategies for PPI implementation often encourage the involvement of diverse stakeholders, not just patients.^20^, ^21^ Indeed, reviews in the mental health field reported that both patients and researchers supported PPI but also expressed feelings of reluctance regarding its use.^18^, ^22^ In addition, recent studies have reported both positive and negative views on PPI by patients, caregivers and mental health service providers.^23^, ^24^ Given that each stakeholder group faces challenges in implementing PPI in research,^20^, ^21^ the perspectives on PPI of a wide variety of stakeholders, not limited to patients or researchers, should be considered. However, in the community mental health service research field, few studies have simultaneously investigated the views and understanding of PPI among patients, caregivers, service providers, researchers and other relevant stakeholders.

Another issue concerning PPI in research is its geographically limited dissemination area. PPI was pioneered in the United Kingdom^25^ and has been promoted mainly in Western countries with democratic cultures, although PPI appears to be universally beneficial for improving research quality. It is noteworthy that most of the studies included in systematic reviews of PPI in mental health research have been conducted in economically developed Western countries.^3^, ^18^, ^22^ In addition, whereas some Asian studies reported stakeholders' views on PPI in the setting of community mental health service delivery,^23^, ^26^ few studies have addressed perspectives on PPI in the context of research in Asia. These facts indicate that little is known about PPI for community mental health service research outside of Western cultures.

To address these evidence gaps, we conducted focus group interviews on PPI for research in patients, caregivers, service providers, government staff and researchers in Japan. This study aimed to qualitatively analyse multiple stakeholders' perspectives on PPI, including expectations and concerns about the challenges and approaches for implementing PPI in community mental health service research outside of Western countries.

## 2 MATERIALS AND METHODS

### 2.1 Research design overview

Face‐to‐face focus group interviews of multiple stakeholders were conducted on 9 February 2020, to assess perspectives on PPI. The focus group method was selected since it was expected that group dynamics would provide diverse perspectives on PPI through discussions with others in Japan where PPI was not disseminated. PPI in mental health services research involves not only patients and researchers but also other stakeholders. Therefore, this study interviewed five groups, respectively, comprised of patients, caregivers, service providers, government staff members and researchers, about their views on PPI and specific challenges or approaches related to PPI. Data were qualitatively analysed using a data‐driven approach, since it seemed suitable for identifying the patterns of meanings and themes expressed by different stakeholder groups. Qualitative analysis was used to create codes, subthemes, themes and domains based on the research questions. The qualitative analysis was conducted from July 2020 to March 2022. We reported the methodology based on the COnsolidated criteria for REporting Qualitative research (COREQ) checklist.^27^

The research project team included a researcher (coauthor, M. O.) who had experience living with mental illness. He was involved in all stages of the study process and attended almost all research meetings. Specifically, together, we developed the research topics and protocols, including the recruitment and analysis plans, and created research materials such as the interview guide. He also facilitated interviews and took part in data analysis and writing of the manuscript. The authors assert that all procedures contributing to this study complied with the ethical standards of the relevant national and institutional committees on human experimentation and with the Helsinki Declaration of 1975, as revised in 2008. Written informed consent was obtained from all participants who were included in the analysis. All procedures in this study were approved by the Research Ethics Committee of the National Center of Neurology and Psychiatry (No. A2019‐021).

### 2.2 Characteristics of the facilitators

A total of seven facilitators participated in the study, including four coauthors (C. F., M. O., N. Y., S. S.). Four facilitators were female, and the mean age was 40.6 years (SD = 7.8). Five had their PhD degree. In terms of professional background, two facilitators were psychiatrists and two were nurses. One facilitator (M. O.) was not only a psychiatrist but also an expert‐by‐experience by virtue of living with mental illness since before becoming a doctor. The remaining two facilitators had mixed backgrounds in nursing, psychology and social work. Facilitators had worked as researchers or mental health service providers for a mean of 11.1 years (SD = 8.5) and 13.6 years (SD = 8.1), respectively. All had previous experience in group facilitation, and before the focus group interviews, they had received a 1‐h lecture by the research team about the interview process and data collection. Some of the facilitators and participants had known each other before the interviews, but there were no collegial, mentoring or financial relationships between them.

### 2.3 Participants and recruitment

The study recruited participants in five stakeholder groups: patients, caregivers, service providers, government staff and researchers. The eligibility criteria of participants were as follows: (1) required to have experienced mental illness in a community setting, have cared for family members with mental illness, have provided community mental health services, have performed government administrative work regarding community mental health or have researched community mental health; (2) age 20 years or older; and (3) have the capacity to consent to participation in the group interview.

We used several strategies to recruit participants. Regarding patients and caregivers, we asked for referrals from large‐scale patient and family organizations; these included three patient or peer support worker organizations, two family organizations and one nonprofit community mental health organization that has been comanaged by patients and service providers. For service providers, we asked national‐level professional associations for nurses, social workers, occupational therapists and clinical psychologists to refer potential participants. Regarding government officials, groups of local government staff focusing on public health or community mental health services were contacted to solicit potential participants. In addition, we directly asked mental health officials at the Ministry of Health, Labour and Welfare of Japan to participate in the interviews. For researchers, we contacted nine academic societies that studied community mental health services and that were registered in lists of Japanese academic organizations.^28^, ^29^ We also contacted the authors of original articles identified using PubMed and Ichushi (Japanese academic database) with the keywords (‘community’ OR ‘recovery’) AND (‘psychiatr*’ OR ‘mental illness’) AND (Japan). When recruiting participants, we asked them to provide their personal perspectives on PPI, rather than views that reflected those of their organizations.

During the aforementioned recruitment process, a total of 45 potential participants were initially contacted. However, eight participants (seven patients and one service provider) could not attend the focus group interviews due to health conditions or issues related to COVID‐19. Thirty‐seven potential participants were given a full description of the study and the ethical issues involved. Finally, all 37 participants agreed to participate and were included in the analysis (patients = 6, caregivers = 5, service providers = 7, government staff = 5 and researchers = 14). Table 1 shows the characteristics of the participants. Most patients and service providers were male. In all stakeholder groups, the mean age was the mid‐40s.

**Table 1:** Characteristics of the participants in each stakeholder group.

	Patients (*n* = 6)		Caregivers (*n* = 5)		Service providers (*n* = 7)		Government staff (*n* = 5)		Researchers (*n* = 14)	
Sex										
Female	0	0.0%	3	60.0%	1	14.3%	2	40.0%	6	42.9%
Male	6	100.0%	2	40.0%	6	85.7%	3	60.0%	8	57.1%
Age (mean, SD)	43.5	7.8	48.6	20.3	46.4	8.9	45.8	10.3	46.1	8.1
Diagnosis^a^										
Schizophrenia	4	66.7%	4	80.0%						
Depression	1	16.7%	1	20.0%						
Bipolar disorder	1	16.7%	0	0.0%						
Duration of illness^a^ (mean, SD)	19.0	4.3	17.4	9.0						
Professional background										
Doctor					0	0.0%	2	40.0%	5	35.7%
Nurse					2	28.6%	1	20.0%	4	28.6%
Social worker					1	14.3%	2	40.0%	3	21.4%
Occupational therapist					2	28.6%	0	0.0%	1	7.1%
Psychologist					2	28.6%	0	0.0%	1	7.1%
Years working as a service provider (mean, SD)					21.4	8.2				
Years working as a government staff (mean, SD)							16.8	12.4		
Years working as a researcher (mean, SD)									17.7	10.0

^a^ For caregivers, the family member's diagnosis and duration of illness.

### 2.4 Data collection

The focus group interviews with each stakeholder were conducted in conference rooms in Tokyo. Before the interview, the first author and the coauthor who had experience with mental illness presented an overall description of PPI and the aim of the project. The participants were then assigned to groups of stakeholders with the same background (e.g., patient group, caregiver group). The participants with a research background were divided into three groups due to the sample size (*n* = 14). A total of seven focus group interviews were performed.

In each stakeholder group, the facilitator performed the interview using a uniform and semi‐structured interview guide (File S1). Participants were asked to discuss two research questions: the first was ‘What do you think about patients/caregivers and researchers working together on research? (What do you think about PPI?)’, and the second was ‘If patients/caregivers and researchers conduct research together, what are the specific challenges or approaches (Are there specific challenges or approaches to PPI?)’. Discussion of each question lasted for around 50 min. Audio recordings were made of all interviews. The facilitators also wrote a summary of participants' discussion points on a whiteboard during each interview, and the contents of the whiteboard were recorded as photographs. We also collected notes that participants took during the interviews.

### 2.5 Data analysis

In the qualitative analysis, we created codes, subthemes, themes and domains based on the data‐driven approach. We performed the coding process by referring to Braun and Clarke's^30^ guidelines. During the coding process, themes were identified at the semantic (i.e., explicit) level, and the data were interpreted verbatim or as literally as possible. In addition, we received supervision from a qualitative research expert (Y. M.) during the analysis.

First, five authors (M. A., M. I., S. Y., T. K. and T. S.) carefully read all interview transcripts. Then, at least two of the five authors reread the transcripts of the stakeholder group to which they were assigned. They independently extracted the data corresponding to the two research questions in each stakeholder group. Since many data extracts consisted of two or three sentences, we also created summaries as long as they did not change the meaning. Finally, a total of 725 summary data extracts were made from the transcripts of all stakeholder groups.

Second, six authors (M. A., M. I., M. O., S. Y., T. K. and T. S.) thoroughly discussed tentative codes, defined as summary data extracts containing common content. Then, three authors (M. A., S. Y. and T. K.) jointly generated initial codes by revising the tentative codes using Microsoft Excel. We also recorded which stakeholder group provided the data at the code level.

Third, three authors (M. A., S. Y. and T. K.) jointly analysed subthemes and themes. They collected relevant codes to generate potential sub‐themes and gathered relevant subthemes into potential themes. Whiteboard photographs and the participants' notes were used as supplemental information to define the subthemes and themes. In addition, the authors created four domains that encompassed similar themes based on the research questions. The definitions and labels of the subthemes, themes and domains were determined by thorough discussions among the three authors to identify the characteristics of each classification. Quotations were selected by the first author to represent each theme. During the analysis process, coding and classification of the subthemes, themes and domains were performed over several iterations.

### 2.6 Trustworthiness

After the main analysis, all coauthors except one (N. Y.) checked the results to ensure that they addressed the research questions and that the terminology was suitable. We also shared all the results with the participants to determine whether they agreed with the results. Finally, Inter‐coder agreement based on Krippendorff's alpha using the codes and themes was determined by a coauthor (N. Y.), who was not involved in the analysis.

## 3 RESULTS

The qualitative analysis resulted in four major domains corresponding to the two research questions: ‘Positive views and expectations regarding PPI’ and ‘General concerns about PPI’ for Research question 1, and ‘Specific issues regarding the implementation of PPI’ and ‘Approaches to PPI implementation’ for Research question 2. These four domains included 11 themes and 33 subthemes established based on the initial 121 codes. The Krippendorff's alpha value for inter‐coder agreement was 0.87. All labels and detailed definitions of domains, themes and subthemes are shown in Table S1.

### 3.1 Domain 1: Positive views and expectations regarding PPI

Table 2 shows the results of the first domain. This domain included the three themes related to positive and supportive views of PPI in a mental health service research setting, improvement in the quality and culture of research and services, and the growth opportunities for both patients/caregivers and researchers.

**Table 2:** Results of thematic analysis, ‘Positive views and expectations regarding PPI’ (Domain 1): What do you think about PPI? (Question 1)

Subtheme			Code	P	C	S	G	R
*Theme 1: A positive perspective on addressing PPI*								
1	Positive views regarding PPI and conveying its value	1	Welcome collaboration between patients and researchers.	✓	✓			✓
		2	PPI should be addressed as a matter of course.	✓	✓		✓	✓
		3	Conveying the value of PPI while simultaneously addressing PPI.					✓
2	Feasibility of PPI in mental health services	4	Addressing PPI in mental health service research appears to be more feasible than in other areas since mental health service is research on interpersonal services.			✓		
3	Ease of collaborating in a research setting	5	An equal relationship in a research setting may be easier to build compared to a clinical setting.				✓	
		6	More interesting collaborations in research than in government activities.	✓				
*Theme 2: Expectations for improving the quality and culture of research and services*								
4	Reflecting the perspectives of patients and caregivers in research	7	PPI will help reflect the voices of patients and caregivers in the research questions.		✓		✓	✓
		8	PPI will help set exposures and outcomes that reflect patients' perspectives.				✓	✓
		9	PPI will help create a questionnaire that reflects patients' perspectives.					✓
		10	PPI will help select data collection methods that reflect patients' perspectives.		✓			✓
5	Improving the quality of research and an open research culture	11	PPI will help select outcome measures or scales.		✓			
		12	PPI will lead to preventing bias in analyses.		✓	✓		✓
		13	PPI will lead to increased sophistication in interview methods.	✓				✓
		14	PPI will improve research ethics.		✓		✓	
		15	PPI will make research more practical and empathetic.		✓		✓	✓
		16	PPI will change the culture of research.			✓	✓	
		17	PPI will make the research process more transparent.				✓	
		18	Dissemination of PPI will increase the number of people, facilities and areas participating in research.					✓
6	Effective and broad dissemination of research findings	19	PPI will enable patients and caregivers to verbalize and communicate their intentions, when disseminating the results of research.		✓			
		20	PPI will enable us to inform the public about issues in the mental health field.	✓	✓		✓	✓
		21	PPI will provide more opportunities for patients to present their work.				✓	✓
		22	A joint presentation in the context of PPI will increase the social impact.					✓
7	Improving the quality of services	23	Improving the quality of services.	✓				
*Theme 3: Expectations of growth opportunities*								
8	Opportunities for patients and caregivers to experience research	24	PPI is an opportunity for patients and caregivers to become more familiar with research.	✓	✓			
		25	PPI will be an opportunity for the patients/caregivers and researchers to come closer together.		✓			
9	Empowerment of patients	26	Patients are empowered by participating in research as PPI.	✓			✓	
		27	PPI will increase opportunities to benefit from the patient's experience.	✓			✓	✓
		28	PPI will serve as an opportunity for patients' social participation and thus reduce stigma.				✓	
10	Opportunities for researchers to reconsider their work	29	PPI will help researchers reconsider their overall research and viewpoints.					✓
		30	PPI will help researchers review their research methods.					✓
		31	PPI will help researchers have another look at their own behaviour.	✓				✓
		32	PPI will help researchers rethink the terminology that they use.	✓		✓	✓	✓

Abbreviations: C, caregiver; G, government staff; P, patient; PPI, patient and public involvement; R, researcher; S, service provider.

***Theme 1: A positive perspective on addressing PPI***

This theme consisted of three subthemes: positive views regarding PPI and the importance of conveying its value to stakeholders, the feasibility of PPI in mental health services and the ease of building collaboration in a research setting. Overall, participants recognized the importance of implementing and promoting PPI. They also considered that implementing PPI in the setting of research would be easier than in the settings of clinical work and administrative activity.

*Since mental health service is an interpersonal service, PPI research is maybe highly feasible*. (A service provider)

*It might be easier to perform collaborative work in a research setting than in a clinical relationship between service providers and recipients…*. (A government staff)

***Theme 2: Expectations for improving the quality and culture of research and services***

This theme included four subthemes: reflecting the perspectives of patients and caregivers in research, improving the quality and culture of research, disseminating research findings and improving the quality of services. In summary, the participants believed that PPI would enhance research and service and promote open research culture.

*….There are many factors that affect quality of life. When I thought about the value hierarchy of factors and about which of these factors should be prioritized for the outcome of the research, In this context, PPI is important*. (A researcher)

***Theme 3: Expectations of growth opportunities***

This theme comprised three subthemes related to the expectation that PPI will result in growth opportunities for patients, caregivers and researchers. The participants acknowledged that PPI resulted in an opportunity for patients and caregivers to learn about research and become empowered by research involvement. PPI was also seen as an opportunity for researchers to gain insight into their own research and behaviours.

*We can be empowered by our involvement in research*. (A patient)

*We often build walls between us and medical and government staff… It is great to have a place and opportunity to collaborate and interact with them through research*. (A caregiver)

### 3.2 Domain 2: General concerns about PPI

The second domain consists of two themes (Table 3): overall concerns about PPI in research, including excessive expectations, and burdens of PPI.

**Table 3:** Results of thematic analysis, ‘General concerns about PPI’ (Domain 2): What do you think about PPI? (Question 1).

Subtheme			Code	P	C	S	G	R
*Theme 4: Excessive expectations of PPI in psychiatry or Japanese culture*								
11	Concern that essential, non‐PPI research is not being promoted, which increases tokenistic PPI	33	Researchers should promote necessary research regardless of PPI.	✓				✓
		34	Patient‐driven research is essential without excessive concerns about PPI.	✓	✓		✓	✓
		35	There is a concern about an increase in research involving tokenistic PPI.		✓		✓	✓
12	Uniqueness of indicators and goals in psychiatry	36	PPI will not solve the problem that mental illness has no clear indicators (e.g., biomarkers).					✓
		37	Only PPI will not create high‐quality evidence because the goal of treating mental illness is not cure, but inclusion of patients in society.					✓
		38	PPI research should consider non‐quantitative evidence due to the nature of psychiatry.					✓
13	Concerns that PPI will bring few changes and benefits in Japanese culture	39	We need to anticipate whether PPI will play a significant role in the future.		✓			✓
		40	Even if PPI is implemented, the findings will not be used in services due to the characteristics of Japanese culture.					✓
		41	PPI in research will not change our society.		✓			
		42	The benefits of PPI may not be worth the efforts of patients and researchers.					✓
*Theme 5: Burdens of PPI and unwillingness to collaborate in research*								
14	Patient and caregiver burdens and unwillingness to collaborate in research	43	Patients and caregivers may be unwilling to cooperate in PPI research due to concerns about stigmatization by researchers.		✓			
		44	PPI will result in psychological burdens on patients.					✓
		45	We have never met a patient who wanted to collaborate in research.			✓		
		46	Some patients are reluctant to collaborate in research since they are unfamiliar with it.		✓			
15	Burdens of collaboration on researchers	47	Researchers find it difficult to define research themes based on the interests of patients and caregivers.					✓
		48	Researchers will feel psychological burden as a result of PPI.				✓	
		49	Researchers will be overburdened because they spend a lot of efforts in explaining technical terms and analysis methods to patients and caregivers.					✓
		50	It will take significant effort for researchers to share the findings of papers with patients and caregivers.					✓
		51	Researchers will have difficulty dealing with patient suggestions if these suggestions deviate from existing ethical guidelines or will lead to methodological bias.			✓	✓	
16	Increases in cost and time	52	PPI with mentally ill patients will be more expensive and time‐consuming when compared to other populations.					✓
		53	PPI research will be more expensive and time‐consuming when compared to non‐PPI research.					✓

Abbreviations: C, caregiver; G, government staff; P, patient; PPI, patient and public involvement; R, researcher; S, service provider.

***Theme 4: Excessive expectations of PPI in psychiatry or Japanese culture***

The participants recognized that there are excessive expectations regarding PPI. This theme consisted of the three subthemes. First, there were concerns that research without PPI and patient‐driven research are not sufficiently promoted, which increases tokenistic PPI research. Second, PPI alone is not always sufficient for solving problems with no clear biological indicators of mental illness or for promoting social inclusion. Third, PPI may have less social benefit than expected, particularly in Japanese culture.

*I feel that there are academically interesting studies…. I might not want researchers to be too respectful of patients' opinions about research. It is OK if the research is interesting and beneficial to society…*. (A patient)

*I don't feel that the research actually makes a change that will affect our lives*. (A caregiver)

***Theme 5: Burdens of PPI and unwillingness to collaborate in research***

In this theme, there were three subthemes related to the psychological, physical and financial burdens of PPI on patients and caregivers. The participants were concerned that PPI research might require more money and time than non‐PPI research. These burdens may result in unwillingness to engage in research collaboration among patients, caregivers and researchers.

*For example, it takes a lot of time to explain complex data in a way that makes it clearer for patients and caregivers. Even then, I think they probably don't understand. So, we spend a lot of time dealing with each other….*. (A researcher)

### 3.3 Domain 3: Specific issues regarding PPI implementation

Table 4 shows the results of the third domain. The analysis identified four themes related to problems implementing PPI, including university and academic society systems, selection methods and conflict of interest issues, relationship building, and ambiguous PPI criteria.

**Table 4:** Results of thematic analysis, ‘Specific issues regarding PPI implementation’ (Domain 3): Are there specific challenges or approaches for PPI? (Question 2).

Subtheme			Code	P	C	S	G	R
*Theme 6: Issues with the current system of universities and academic organizations*								
17	Issues with employment, wages and work and research environment	54	No rules for appropriate wages, transportation costs or compensatory days off for patients and caregivers who collaborate with research.	✓	✓			
		55	Issues related to employing a researcher who is an expert by experiences of living with mental illness.				✓	
		56	Difficulty in compensating patients who collaborate with researchers in the current system.					✓
		57	Systemic issues related to the participation of patients in research, such as the availability of identification numbers for researchers (which are necessary for grant application in Japan).					✓
		58	Lack of opportunities for people unaffiliated with universities to attend lectures on research skills and ethics.					✓
18	Problems with evaluation of researchers	59	Research institutes do not value topics that are unlikely to lead to manuscript publication.	✓		✓		✓
		60	Research is socially undervalued if it cannot be published in English.					✓
19	Issues with the academic society system	61	The academic society system imposes requirements for membership.					✓
		62	The academic society system does not allow non‐members to serve as authors (coauthors).		✓			✓
		63	The academic society system does not allow non‐members to access papers.		✓			
*Theme 7: Issues with the selection of patients and caregivers*								
20	Issues regarding required research skills of patients and caregivers and their disease condition	64	Too many research skills are required for patients and caregivers who are new to research.		✓			✓
		65	When writing papers, it is difficult to collaborate with patients due to their lack of academic skills.					✓
		66	Practical difficulties in implementing PPI for some diseases and conditions.		✓		✓	✓
21	Problems with representativeness and conflicts of interest	67	Concerns about whether the views of patients and caregivers who collaborate with research are representative of others with similar problems.		✓		✓	✓
		68	Problems with the process of choosing patients and caregivers who collaborate with research.				✓	✓
		69	Concerns that PPI with only a few patients and caregivers will bias opinions.		✓		✓	✓
		70	Problem that representativeness cannot be guaranteed if the emphasis is on relationships and feasibility.			✓	✓	✓
		71	Need to disclose conflicts of interest with commercial enterprises (e.g., pharmaceutical companies).	✓				✓
		72	Establishing cooperative relationships with patient organizations.	✓			✓	
		73	Need to disclose patients' conflicts of interest if they are the ones conducting the research.	✓				✓
*Theme 8: Issues with relationship building and forming a consensus*								
22	Issues with building partnerships	74	Feasibility of partnerships between researchers and patients/caregivers based on ‘real’ equal relationships.	✓	✓	✓	✓	✓
		75	Unavoidable existence of an authority gradient when a researcher teaches a patient about research.					✓
		76	Difficulties in the quality of relationships with researchers, as subjectively perceived by patients and caregivers regardless of fact.	✓	✓	✓	✓	✓
		77	Patients without research skills feel psychologically defeated in a research setting.	✓	✓			✓
		78	Problems with distinguishing between the people who research and those who are researched.	✓				✓
		79	Problems of researchers having excessive concern and consideration for patients.	✓				
23	Researchers' lack of PPI‐related skills for building a consensus in the face of diverse opinions	80	The opinions of those who are more assertive are likely to be adopted.				✓	✓
		81	Difficulties in forming a consensus when diverse opinions exist.				✓	✓
		82	Problems with diverse views of patients and caregivers not being reflected in the research methods.	✓				✓
		83	Problems with researchers not being adequately trained and lacking PPI‐related skills.					✓
*Theme 9: Issues with ambiguous PPI criteria*								
24	Ambiguity of criteria and definitions of PPI	84	The nature of research should be reconsidered on the basis of PPI.					✓
		85	The rules and criteria that define various PPI should be clarified.	✓		✓	✓	✓
		86	It should be recognized that PPI can involve different levels of research collaboration.					✓
		87	Coproduction research that is more collaborative than PPI should be explored.					✓

Abbreviations: C, caregiver; G, government staff; P, patient; PPI, patient and public involvement; R, researcher; S, service provider.

***Theme 6: Issues with the current system of universities and academic organizations***

Participants recognized the inadequacy of systems for supporting PPI in academic fields, specifically, three subthemes related to issues surrounding employment of patients as researchers, leading to limited access to training courses for research skills and ethics; performance evaluation of researchers; and membership available only to academics.

…*If researchers have to ensure that the results are published as a paper, it may be difficult for patients to participate in the research*. (A service provider)

***Theme 7: Issues with the selection of patients and caregivers***

The analysis found two concerns in the process of selecting patients and caregivers for PPI participation. The first subtheme pertained to the fact that individual research skills or disease status may be priorities in the selection process. The second subtheme concerned the representativeness of patients and caregivers, including problems related to conflicts of interest. If individuals receive funding from a company related to the research topics or belong to an organization that promotes a specific political or social movement related to the research topics, their views may be biased rather than representative.

*The problem is who you invite to participate. I always ask the same patients. I wonder if they are representative of other patients*. (Researcher group)

***Theme 8: Issues with relationship building and forming a consensus***

The theme included two subthemes. The first related to building partnerships between patients/caregivers and researchers; the participants in all groups were concerned about equality in the partnerships, especially given the differences in power between individuals. The second pertained to researchers' lack of PPI‐related skills for properly forming a consensus in the face of diverse opinions of patients and caregivers.

*Equality is very important. I think it is crucial to have a proper discussion in a partnership, but that is the most difficult part of the current situation in Japan*. (A caregiver)

***Theme 9: Issues with ambiguous PPI criteria***

The participants pointed out the need for clear definitions, criteria and rules for the implementation of PPI. Various approaches to research collaboration were also expected.

*If we don't make a rule of PPI that requires us to really listen to patients' voices, this listening will become just an act*. (A government staff)

### 3.4 Domain 4: Approaches to PPI implementation

The fourth domain consisted of two themes related to specific approaches to implementing PPI (Table 5). The first theme pertained to strategies that facilitate mutual understanding with patients and caregivers, while the second concerned the need for a system and guidelines to properly implement PPI in community mental health services research.

**Table 5:** Results of thematic analysis, ‘Approaches to PPI implementation’ (Domain 3): Are there specific challenges or approaches for PPI? (Question 2).

Subtheme			Code	P	C	S	G	R
*Theme 10: Strategies for facilitating mutual understanding in the research process*								
25	Using understandable words and providing an open and detailed explanation of the research process	88	Use of plain language throughout the research process.		✓	✓		✓
		89	Use of terms that demonstrate sensitivity to patients and caregivers.					✓
		90	Enthusiastically communicating with patients and caregivers about PPI.	✓	✓		✓	✓
		91	Explanation of the advantages and disadvantages of PPI.	✓				✓
		92	Explanation of terminology (technical language) in research.		✓			
		93	Explanation of the expected roles of patients and caregivers when working on the research.		✓	✓		✓
		94	Sharing the roles of each stakeholder group based on an understanding of the differences in individual viewpoints.		✓		✓	✓
		95	Explaining the setting and limitations of the study, including the study duration.					✓
		96	Explanation of how to share the results of PPI studies.		✓	✓		
		97	Explaining and confirming the ownership of research results in the early stages of research.					✓
		98	Discussion of presentation contents, publication type and journal language (Japanese or English).					✓
		99	Including the interpretations of both the patients and researchers in publications.					✓
26	Establishing an atmosphere that allows for free exchange of ideas	100	Creating an atmosphere where patients and caregivers can freely express their opinions.		✓		✓	✓
		101	Reasonable accommodation for disclosure of personal information in PPI.				✓	✓
		102	Advance preparation for active exchange of opinions.	✓	✓		✓	✓
		103	For active exchange of opinions, ensuring opportunities to talk only with people who have the same background.		✓			✓
27	Effective team management	104	Management of patient burdens in research work.		✓			✓
		105	Managing relationships among team members, including patients and caregivers.					✓
		106	Establishing how to care for patients when they feel unwell.				✓	✓
		107	Use of communication methods preferred by patients and caregivers.					✓
*Theme 11: Development of systems and guidelines for implementation of PPI*								
28	A system for matching diverse patients/caregivers with research objectives	108	Service providers with a role in connecting researchers and patients.		✓	✓		✓
		109	Trained patients with a role in connecting researchers and patients.					✓
		110	A human resource bank for patients and caregivers to ensure diverse human resources.			✓		✓
		111	A system to match diverse patients and caregivers with research objectives and content.				✓	✓
		112	A system that does not exclude patients who have not been trained in research.				✓	✓
		113	Developing a system that interests researchers and facilitates their participation in a patient‐led study.	✓				✓
29	A system to evaluate PPI	114	A system in which institutions and patients participating in PPI are highly valued.		✓		✓	
		115	A system to evaluate the PPI process after the study is completed.					✓
30	Developing research ethics and guidelines for PPI	122	Developing guidelines for PPI in conjunction with patients and caregivers.		✓			✓
		123	Developing a framework for PPI‐related research ethics.					✓
31	Training for researchers and patients/caregivers	116	Developing training for researchers who can collaborate with patients and caregivers.			✓	✓	✓
		117	Developing training for patients and caregivers on research and analysis.		✓	✓	✓	✓
		118	Developing training for patients and caregivers on ethical issues and personal information.					✓
32	Accumulation of successful experiences and presentation of model cases	119	Identifying patients and caregivers who can serve as role models related to successful PPI.				✓	
		120	Documenting the accumulation of successful PPI experiences.				✓	
33	Strengthening the network and information transmission	121	Strengthening the network and information transmission of patient organizations.	✓				✓

Abbreviations: C, caregiver; G, government staff; P, patient; PPI, patient and public involvement; R, researcher; S, service provider.

***Theme 10: Strategies for facilitating mutual understanding in the research process***

Based on the participants' discussions, three subthemes were found: explaining and discussing the entire research process with patients and caregivers using familiar language; establishing an atmosphere that allows for free, safe and active exchange of ideas; and ensuring an effective team management system to address the aforementioned issues. The participants believed these potential strategies may promote mutual understanding.

*I feel that it is vital to create an atmosphere where patients can talk honestly. Unless they establish such an atmosphere, researchers will not hear patients' true voices*. (A government staff)

***Theme 11: Development of systems and guidelines for implementation of PPI***

In this theme, the analysis identified six subthemes related to promoting and implementing PPI. For example, PPI was expected to be improved by the use of human resource matching to connect people interested in specific research topics, by developing a system to evaluate the process of PPI and by ensuring that people and institutions that participate in PPI are highly valued. It was also considered essential to develop research ethics and guidelines and organize a training system for patients, caregivers and researchers. In addition, successful role models for PPI and strengthening patient organizations were considered potential contributors to facilitating PPI.

*It would be nice if a human resource agency could suggest a person who would be suitable for a particular topic. It would also be nice if they serve as an agent for patients and protect patients from undue disadvantages or burdens*. (A researcher)

*…Researchers can use patient organization networks such as their newsletters. Increased transmission of information to patients results in the patients becoming more equal…. If one side in a power balance is weak, they are weak no matter what they do. I think it is important for patient organizations to have enough resources that researchers may want to utilize*. (A patient)

## 4 DISCUSSION

This study qualitatively analysed the perspectives of multiple stakeholder groups in Japan regarding the implementation of PPI in community mental health service research. The analysis identified domains that reflected positive views and general concerns about PPI, as well as specific issues and approaches concerning PPI implementation. The participants' positive feedback and the high Krippendorff's alpha value appear to indicate the reliability of the results. Some results of this study replicated those of Western studies, while others were unique to Japan. Therefore, we discuss the results in comparison with previous studies.

Positive perspectives and expectations about PPI were confirmed in a Japanese setting. Not surprisingly, numerous Western studies have acknowledged the potential value of PPI in research.^6^, ^7^, ^8^, ^9^, ^10^, ^11^, ^12^, ^13^, ^15^, ^16^, ^17^, ^18^, ^19^ In particular, patients with mental illness tend to seek involvement in research activity.^31^, ^32^ In addition, improvement in the quality of research and services and increased patient empowerment have been reported in other studies.^6^, ^7^, ^8^, ^9^, ^10^, ^11^, ^12^, ^13^, ^15^ These issues appear to be common expectations concerning PPI in research in Western countries and Japan. On the other hand, the optimistic views about PPI in mental health service research settings compared to clinical settings or other research areas may be unique to this study. In routine clinical work in the community mental health field, staff members often face barriers to collaborative work with patients or peer support workers in relationships with an authority gradient.^14^, ^17^, ^33^ The results of this study may have been affected by the participation of service providers and government staff in the interviews.

Overall concerns about PPI were also found. This study identified the potential for excessive expectation including tokenistic PPI and an increased research burden. These concerns were reported in a previous review.^16^, ^17^, ^18^, ^34^ Participants also recognized the value of research without PPI, as well as the limitation that PPI is not a perfect solution for improving their lives or achieving individual treatment goals because the biological mechanisms of mental illness are still unknown. On an international level, researchers have begun debating the balance between the costs and benefits of PPI.^35^ It may be necessary to recognize that an overemphasis on PPI may result in enforcement of its use and the undermining of freedom and success of research. Also, negative views on PPI as a solution for current issues may have been due to the Japanese context. The participants were concerned that even if PPI yields good evidence, it may not improve the lives of people with mental illness and their families in Japanese culture. This disappointment appears to be related to Japanese political systems, which have lagged far behind those of other countries in the implementation of evidence‐based policymaking.^36^ In other words, the findings might indicate that, regardless of whether PPI is implemented in research settings, stakeholders in Japan are likely to have a common perception that research evidence does not influence community mental health policy. Considering that the original goal of PPI was to reflect patients' voices in medical and social care policies,^25^ this issue should be highlighted when PPI is promoted worldwide.

Several specific issues regarding PPI implementation in the real world were also identified in this analysis. Previous studies have frequently reported issues related to the following: insufficient systems for supporting PPI and the representativeness and conflicts of interest of patients and caregivers involved in research, partnership and consensus‐building.^10^, ^16^, ^17^, ^18^, ^19^, ^37^ In particular, establishing systems to support PPI, including the employment of researchers who are experts by experiencing living with mental illness, is a common challenge even in European countries.^38^, ^39^ Japan may have greater difficulty with this problem than other countries, because in general, only people affiliated with research institutes and universities can apply for government research funds. Also, many Japanese academic societies allow only their members to read articles that they have published and to present study findings in a conference or a journal. Therefore, many patients and caregivers have little opportunity to disseminate PPI research as a copresenter or coauthor in Japanese academic communities. Similary, the issue of partnership is more serious in Japanese culture than in other cultures. Japanese society historically developed as an authoritarian culture and paternalistic attitudes persist. Indeed, all the stakeholder groups were concerned about partnerships in the presence of power gradients. While partnership issues stemming from power dynamics often arise when a patient and a researcher work together in the western country,^40^ this appears to be a barrier that should be addressed particularly closely when implementing PPI in Japan.

The analysis found multiple approaches to PPI implementation, including the following: sharing information on roles and research processes; fostering an empathic atmosphere; developing training, guideline and ethics systems; and empowering patient organizations and human capital matching systems to connect patients/caregivers and researchers. Most of these have been reported in previous studies as key approaches to PPI.^10^, ^15^, ^18^, ^39^, ^41^, ^42^ Given the characteristics of Japanese culture, creating a free, safe and empathic atmosphere in research activities is imperative in the community mental health field.^18^ In general, people learn more effectively in a psychologically safe space.^43^ In addition, empathic communication skills make it more likely that patients will feel safe to express their own ideas.^44^ A previous study on PPI in mental health research emphasized that the knowledge and experiences of team members were shared most effectively when they felt psychologically and physically safe in open and trusting environments.^2^ Given the power gradient between researchers and patients/caregivers, establishing a free, safe and empathetic atmosphere may be particularly important in PPI. This importance may apply to other countries as well. In summary, substantial changes to research procedures, academic systems and attitudes of individual researchers are required to implement PPI in Japan and worldwide.

### 4.1 Strengths and limitations of the study

The strengths of this study are twofold. First, several stakeholder groups participated in the interviews. The study findings were based not only on the opinions of patients and researchers but also on those of caregivers, service providers and government staff. The second strength was the systematic recruitment process of potential participants. This may have contributed to the avoidance of selection bias.

We recognize two major limitations of this study. First, seven potential participants in the patient group did not attend the interviews due to health conditions or the COVID‐19 pandemic. Therefore, there were fewer participants in the patient group than we expected. Second, all participants in the patient group were males. While this result is coincidental, and it is unclear whether the views on PPI were affected by sex, our findings did not reflect the perceptions of female patients.

## 5 CONCLUSION

This study explored views on the implementation of PPI in community mental health services research among stakeholders who included patients, caregivers, service providers, government staff and researchers. While participants in multiple stakeholder groups had supportive views on the implementation of PPI, they also had overall concerns (e.g., burden and token use of PPI) and specific issues with PPI (e.g., representativeness and partnership). Furthermore, the participants believed that substantial changes in academic systems and the attitudes of individual researchers were necessary to implement PPI in Japan. This study replicated most of the barriers and approaches to PPI that were previously reported by qualitative research in Western counties. However, utilization of evidence produced by PPI research and partnership in the PPI process may be particularly serious issues in Japan. Future PPI studies need to carefully address solutions that fit each culture.

## AUTHOR CONTRIBUTIONS

Sosei Yamaguchi, Makiko Abe, Momoka Igarashi, Makoto Ogawa, Naonori Yasuma, Sayaka Sato and Chiyo Fujii, conceived and designed the study. Sosei Yamaguchi, Makiko Abe, Momoka Igarashi, Makoto Ogawa, Naonori Yasuma, Sayaka Sato and Chiyo Fujii were responsible for data acquisition. Sosei Yamaguchi, Momoka Igarashi and Yuki Miyamoto planned the analysis. Sosei Yamaguchi, Takayuki Kawaguchi, Makiko Abe, Momoka Igarashi and Takuma Shiozawa analysed data. Makoto Ogawa and Naonori Yasuma supported the analysis. Chiyo Fujii provided administrative and technical support. Sosei Yamaguchi contributed to the writing of the draft manuscript. All authors contributed to the interpretation of the results and writing of the manuscript, and approved the final version of the manuscript.

## CONFLICTS OF INTEREST

The authors declare no conflicts of interest.

## ETHICS STATEMENT

This study was approved by the Research Ethics Committee at the National Centre of Neurology and Psychiatry (A2019‐21). The authors assert that all procedures contributing to this study comply with the ethical standards of the relevant national and institutional committees on human experimentation and with the Helsinki Declaration of 1975, as revised in 2008.

## Supporting information

Supporting information.

## Data Availability

Not all data are freely accessible because no informed consent was given by the participating agencies for open data sharing. However, the data are available from the corresponding author on reasonable request, following approval by the Research Ethics Committee at the National Center of Neurology and Psychiatry.
