# Supplementary material for: Multiple stakeholders' perspectives on patient and public involvement in community mental health services research: A qualitative analysis
Source: Health Expect. 2022 Jun 3;25(4):1844–60. doi: 10.1111/hex.13529 (PMC9327805; doi:10.1111/hex.13529)
Supplement: Supplementary file 1 — Supporting information. [file HEX-25--s001.docx]

***Online supplementary file***

**Focus group interview guide**

**09/02/2020**

**Note**

The purpose of this group interview is to consider or discuss the ways that patients/caregivers and researchers can conduct research studies together (patient and public involvement: PPI). Therefore, the group interview does not aim to identify what methods are right or wrong, or what research is most or least important.

Rule 1: Each statement should be no longer than three minutes, if possible.

Rule 2: Please do not interrupt others when they are speaking.

Rule 3: Please do not interrupt when others are speaking.

Rule 4: The facilitator will write a summary of your statements on the whiteboard to help you understand each other’s comments.

Remark: An audio recording is being made of the interview. You are free to take notes, but they will be collected after the interview.

**Question 1 (50 min):** **What do you think about PPI?**

**What do you think about patients/caregivers and researchers working together on research?**

**Example of sub-questions**

1. Do you think there is anything good (advantages) or bad (disadvantages) about patients/caregivers collaborating with researchers on research?
2. Would your willingness to collaborate change depending on the research theme or method? If so, please tell us the reason.

Remark: We would very much like to hear what you have to say. Any ideas would be welcome.

This question is not about what you can/can’t actually do.

**Question 2 (50 min): Are there specific challenges or approaches to PPI?**

**For patients and caregivers**

**If patients/caregivers and researchers were to conduct research together, what would be the specific challenges or approaches, what would be your desired involvement in the research, and what reasonable accommodations would you need from the researcher?**

**For service providers, government staff, and researchers**

**If patients/caregivers and researchers were to conduct research together, what would be the specific challenges or approaches and how would you like patients/families to be involved in your research?**

**Example of sub-questions**

1. If you were working on a patient–researcher collaborative study, which (or all) phases of the study would you want to or could you collaborate on?
2. If you were working on a patient–researcher collaborative study, what would be the difficulties and what systems or improvements would be necessary?

Remark: We would very much like to hear what you have to say. Any ideas would be welcome.

This question is not about what you can/can’t actually do.

**Online Supplementary Table. Results of thematic analysis: Definitions of all domains, themes, and sub-themes**

| **Domain** | **Definition** |  | **Theme** | **Definition** |  | **Sub-theme** | **Definition** |
| --- | --- | --- | --- | --- | --- | --- | --- |
| **1.**  **Positive views and expectations regarding PPI** | Positive and supportive  views of PPI, for instance that psychiatric research tends to be more likely to engage in collaborative and equal relationships, that PPI improves the quality of research and services, and that PPI provides an opportunity for interaction between patients/caregivers and researchers | 1 | **A positive perspective on addressing PPI** | Positive and supportive views on PPI and optimistic views that it is easier to implement PPI and to build equal relationships in mental health service research than in other research fields and clinical work. | 1 | **Positive views regarding PPI and conveying its value** | Positive views on the implementation of PPI, and views that it is important to convey the value of PPI while simultaneously addressing its problems |
|  |  |  |  |  | 2 | **Feasibility of PPI in mental health services** | The opinion that PPI may be more feasible in mental health services than in other fields, since mental health service research involves research on interpersonal services |
|  |  |  |  |  | 3 | **Ease of collaborating in a research setting** | Expectations that building equal relationships and viable collaborations are easier in a research setting compared to a clinical work setting or an administrative activity setting |
|  |  | 2 | **Expectations for improving the quality and culture of research and services** | Expectations that PPI will increase the quality of research methods, will make research findings more practical and accessible by integrating the perspectives of patients and caregivers, and will lead to transparency in the research process and culture | 4 | **Reflecting the perspectives of patients and caregivers in research** | Expectations that PPI will reflect the perspectives of patients and caregivers in the research, and will, for example, influence research questions or questionnaire design |
|  |  |  |  |  | 5 | **Improving the quality of research and an open research culture** | Expectations that PPI can contribute to improving the quality of research and the utility of findings, including the selection of outcome measures, data collection methods, analysis, ethics, transparency, and accessibility to research. PPI is also expected to change the research culture itself. |
|  |  |  |  |  | 6 | **Effective and broad dissemination of research findings** | Expectations that the joint presentation of research findings that reflect patients’ and caregivers’ views will have a broad and significant social impact |
|  |  |  |  |  | 7 | **Improving the quality of services** | Expectations that PPI will improve the quality of services |
|  |  | 3 | **Expectations of growth opportunities** | Expectations that PPI will enable interactions between patients/caregivers and researchers, resulting in an opportunity for patients and caregivers to learn about the research and be empowered, and an opportunity for researchers to gain insight into their own research and behaviours | 8 | **Opportunities for patients and caregivers to experience research** | Expectations that PPI will be an opportunity for patients and caregivers to learn about the research and to become closer to the researchers |
|  |  |  |  |  | 9 | **Empowerment of patients** | Expectations that PPI will increase the number of opportunities for patient activities, leading to their own empowerment and a reduction in stigma |
|  |  |  |  |  | 10 | **Opportunities for researchers to reconsider their work** | Expectations that PPI will help researchers reconsider or review their research methods, position, behaviours, and terminology |

| **Online Supplementary Table, contd.** | | | | | | | |
| --- | --- | --- | --- | --- | --- | --- | --- |
| **Domain** | **Definition** |  | **Theme** | **Definition** |  | **Sub-theme** | **Definition** |
| **2.**  **General concerns about PPI** | Overall concerns about PPI research, including the fact that some research does not require PPI, that PPI is not a solution to everything, that PPI is implemented in research in a tokenistic manner, and that PPI will increase physical and psychological burdens on patients and researchers | 4 | **Excessive expectations of PPI in psychiatry or Japanese culture** | Concerns that the effectiveness of PPI research is overestimated (PPI may not always solve particular problems in psychiatry or social problems in Japanese culture) and non-PPI research is not promoted | 11 | **Concern that essential, non-PPI research is not being promoted, which increases tokenistic PPI** | Concern that research is too limited to PPI, which increases tokenistic PPI, and researchers and patients should also promote research that they feel is necessary |
|  |  |  |  |  | 12 | **Uniqueness of indicators and goals in psychiatry** | View that only PPI will not solve the problem that psychiatry has no clear, measurable, biological indicators, and that the goals of psychiatric treatment are not curing patients but facilitating their social inclusion |
|  |  |  |  |  | 13 | **Concerns that PPI will bring few changes and benefits in Japanese culture** | Concerns that the results of PPI may not always provide social changes or benefits due to the cultural characteristics of Japanese society, and that people may have excessive expectations of PPI |
|  |  | 5 | **Burdens of PPI and unwillingness to collaborate in research** | Concerns that PPI may require more money and time than non-PPI research, and that patients and researchers may face psychological, physical, and financial burdens and unwillingness to participate in PPI research | 14 | **Patient and caregiver burdens and unwillingness to collaborate in research** | Concerns that some patients and caregivers may be unwilling to participate in PPI due to unfamiliarity with research or researcher’s attitudes |
|  |  |  |  |  | 15 | **Burdens of collaboration on researchers** | The view that incorporating patients’ opinions into research is a separate issue from whether a study is feasible and validated or not, and therefore researchers feel burdened by collaboration |
|  |  |  |  |  | 16 | **Increases in cost and time** | Concern that PPI may be physically, temporally, and financially burdensome for both researchers and patients |

| **Online Supplementary Table, contd.** | | | | | | | |
| --- | --- | --- | --- | --- | --- | --- | --- |
| **Domain** | **Definition** |  | **Theme** | **Definition** |  | **Sub-theme** | **Definition** |
| **3.**  **Specific issues regarding the implementation of PPI** | Practical and specific issues that may occur when PPI is implemented in the current academic environment. This includes problems with university and academic society systems, selection methods, relationship issues, conflicts of interest, and ambiguous PPI criteria | 6 | **Issues with the current system of universities and academic organizations** | Problems related to inadequate systems to support PPI, such as employment issues leading to limited access to training courses, performance evaluation of researchers, and limited opportunities for patients and caregivers to access academic society | 17 | **Issues with employment, wages, and work and research environment** | Issues with the work environment, including employment and wages related to patients and caregivers who participate in research, take research and ethics training courses, and obtain researcher identification numbers in Japan |
|  |  |  |  |  | 18 | **Problems with evaluation of researchers** | Topics that unlikely lead to manuscript publication are unlikely to be adopted, since researchers are evaluated and can gain social recognition based on their publications |
|  |  |  |  |  | 19 | **Issues with the academic society system** | Problems with the current academic society system making it difficult for patients and caregivers to join academic societies, present their research, and publish manuscripts |
|  |  | 7 | **Issues with the selection of patients and caregivers** | Issues with the research skills and disease status of collaborating patients and caregivers, these individuals’ representativeness, and biased views that arise if patients belong to or are funded by organizations that have conflicts of interest with the research topic | 20 | **Issues regarding required research skills of patients and caregivers and their disease condition** | Collaborating patients/caregivers may be required to have many research skills, resulting in their reluctance to participate in research. In addition, these skills may be affected by cognitive and other symptoms of the disease |
|  |  |  |  |  | 21 | **Problems with representativeness and conflicts of interest** | Challenges related to the methods of selecting patients and caregivers participating in research, the representativeness of their views, and the fact that conflicts of interest leading to biased views arise if individuals participating in PPI research receive funding from a company related to the research topic, or if they belong to an organization that promotes a specific political or social movement related to the research topic |
|  |  | 8 | **Issues with relationship building and forming a consensus** | Issues with building a partnership between patients/caregivers and researchers and consolidating diverse opinions under power gradients | 22 | **Issues with building partnerships** | Power gradients and challenges in building equal relationships between patients/caregivers and researchers |
|  |  |  |  |  | 23 | **Researchers’ lack of PPI-related skills for building a consensus in the face of diverse opinions** | Researchers are not trained and lack skills in how to contact, communicate with, explain their research to, and incorporate the views of diverse patients and caregivers and form a consensus that takes into account a wide variety of patient and caregiver opinions |
|  |  | 9 | **Issues with ambiguous PPI criteria** | Views that the definitions, criteria, and implementation rules of PPI should be clarified | 24 | **Ambiguity of criteria and definitions of PPI** | View that the definitions, criteria, and implementation rules of PPI must be clarified, and that there is a need to allow for flexible and various forms of PPI based on the rules |

| **Online Supplementary Table, contd.** | | | | | | | |
| --- | --- | --- | --- | --- | --- | --- | --- |
| **Domain** | **Definition** |  | **Theme** | **Definition** |  | **Sub-theme** | **Definition** |
| **4.**  **Approaches to PPI implementation** | Specific approaches to the implementation of PPI: a research promotion system that provides reasonable accommodation for patients and caregivers, including explanations that are easy for them to understand; establishing agreements on the handling of research results; creating a tolerant atmosphere; establishing a system of personnel matching, guidelines, and training; and expanding the network of patients’ organizations that researchers may want to utilize. | 10 | **Strategies for facilitating mutual understanding in the research process** | Explaining and discussing the entire research process with patients and caregivers using familiar language in an atmosphere and a team management system that allow for free, safe, and active exchange of ideas | 25 | **Using understandable words and providing an open and detailed explanation of the research process** | Using clear language, enthusiastically communicating with patients and caregivers about PPI, its purpose, research periods, the role of each stakeholder, advantages and disadvantages, terminology, and interpretation and use of results |
|  |  |  |  |  | 26 | **Establishing an atmosphere that allows for free exchange of ideas** | Establishing an atmosphere that allows for free, safe, and active exchange of ideas, including advance preparation for such exchange, enabling disclosure of personal information, and ensuring opportunities to talk only with people who have the same background |
|  |  |  |  |  | 27 | **Effective team management** | Team management in the research team, including patient burdens, relationships, and how to care for patients when they feel unwell |
|  |  | 11 | **Development of systems and guidelines for implementation of PPI** | Establishing systems that facilitate PPI, including human resource matching to connect people interested in specific research topics, a system to evaluate the content and process of PPI, research ethics and guidelines for PPI, training of patients, caregivers, and researchers, role models for PPI, and strengthening and expanding the network and information transmission of patient organizations | 28 | **A system for matching diverse patients/caregivers with research objectives** | Establishment of a system for matching diverse patients and caregivers to the objectives of each research including a patient-led study; the system includes a human resource bank and collaboration with trained patients and service providers |
|  |  |  |  |  | 29 | **A system to evaluate PPI** | Developing a system to evaluate the content and process of PPI and to ensure that people and institutions that participate in PPI are highly valued |
|  |  |  |  |  | 30 | **Developing research ethics and guidelines for PPI** | Developing research ethics and guidelines for PPI in conjunction with patients and caregivers |
|  |  |  |  |  | 31 | **Training for researchers and patients/caregivers** | Developing programs to train researchers, patients, and caregivers to implement proper PPI |
|  |  |  |  |  | 32 | **Accumulation of successful experiences and presentation of model cases** | Accumulating successful PPI experiences, such as documenting the PPI research process and identifying patients and caregivers who can serve as role models |
|  |  |  |  |  | 33 | **Strengthening the network and information transmission** | Strengthening and expanding the network and information transmission of patient organizations that researchers may want to utilize. |
